# Polymeric reinforcements for cellularized collagen-based vascular wall models: influence of the scaffold architecture on the mechanical and biological properties

**DOI:** 10.3389/fbioe.2023.1285565

**Published:** 2023-11-16

**Authors:** Nele Pien, Dalila Di Francesco, Francesco Copes, Michael Bartolf-Kopp, Victor Chausse, Marguerite Meeremans, Marta Pegueroles, Tomasz Jüngst, Catharina De Schauwer, Francesca Boccafoschi, Peter Dubruel, Sandra Van Vlierberghe, Diego Mantovani

**Affiliations:** ^1^ Laboratory for Biomaterials and Bioengineering, Canada Research Chair Tier I for the Innovation in Surgery, Department of Min-Met-Materials Engineering and Regenerative Medicine, CHU de Quebec Research Center, Laval University, Quebec City, QC, Canada; ^2^ Polymer Chemistry and Biomaterials Group, Centre of Macromolecular Chemistry, Department of Organic and Macromolecular Chemistry, Ghent University, Ghent, Belgium; ^3^ Faculty of Veterinary Medicine, Department of Translational Physiology, Infectiology and Public Health, Ghent University, Merelbeke, Belgium; ^4^ Laboratory of Human Anatomy, Department of Health Sciences, University of Piemonte Orientale “A. Avogadro”, Novara, Italy; ^5^ Department of Functional Materials in Medicine and Dentistry, Institute of Biofabrication and Functional Materials, University of Würzburg and KeyLab Polymers for Medicine of the Bavarian Polymer Institute (BPI), Würzburg, Germany; ^6^ Biomaterials, Biomechanics and Tissue Engineering Group, Department of Materials Science and Engineering, Universitat Politècnica de Catalunya, Barcelona, Spain

**Keywords:** vascular wall model, cellularized collagen, polymeric reinforcement, solution electrospinning, melt electrowriting, 3D printing

## Abstract

A previously developed cellularized collagen-based vascular wall model showed promising results in mimicking the biological properties of a native vessel but lacked appropriate mechanical properties. In this work, we aim to improve this collagen-based model by reinforcing it using a tubular polymeric (reinforcement) scaffold. The polymeric reinforcements were fabricated exploiting commercial poly (ε-caprolactone) (PCL), a polymer already used to fabricate other FDA-approved and commercially available devices serving medical applications, through 1) solution electrospinning (SES), 2) 3D printing (3DP) and 3) melt electrowriting (MEW). The non-reinforced cellularized collagen-based model was used as a reference (COL). The effect of the scaffold’s architecture on the resulting mechanical and biological properties of the reinforced collagen-based model were evaluated. SEM imaging showed the differences in scaffolds’ architecture (fiber alignment, fiber diameter and pore size) at both the micro- and the macrolevel. The polymeric scaffold led to significantly improved mechanical properties for the reinforced collagen-based model (initial elastic moduli of 382.05 ± 132.01 kPa, 100.59 ± 31.15 kPa and 245.78 ± 33.54 kPa, respectively for SES, 3DP and MEW at day 7 of maturation) compared to the non-reinforced collagen-based model (16.63 ± 5.69 kPa). Moreover, on day 7, the developed collagen gels showed stresses (for strains between 20% and 55%) in the range of [5–15] kPa for COL, [80–350] kPa for SES, [20–70] kPa for 3DP and [100–190] kPa for MEW. In addition to the effect on the resulting mechanical properties, the polymeric tubes’ architecture influenced cell behavior, in terms of proliferation and attachment, along with collagen gel compaction and extracellular matrix protein expression. The MEW reinforcement resulted in a collagen gel compaction similar to the COL reference, whereas 3DP and SES led to thinner and longer collagen gels. Overall, it can be concluded that 1) the selected processing technique influences the scaffolds’ architecture, which in turn influences the resulting mechanical and biological properties, and 2) the incorporation of a polymeric reinforcement leads to mechanical properties closely matching those of native arteries.

## Graphical Abstract

**Figure:**
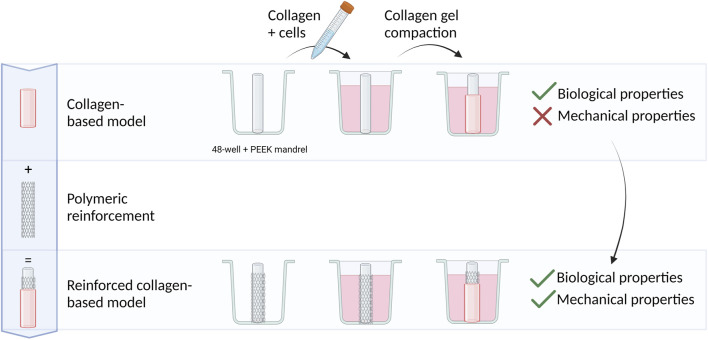
Created with BioRender.com.

## Highlights

▪Solution electrospinning, 3D printing and melt electrowriting were benchmarked against the non-reinforced collagen-based model
▪Solution electrospinning, 3D printing and melt electrowriting resulted in differences in fiber alignment, diameter and pore size
▪The scaffolds’ architecture influences their mechanical and biological properties
▪Polymeric reinforcements lead to improved mechanical properties compared to the non-reinforced collagen-based model

## 1 Introduction

Although tissue engineered blood vessels (TEBV) have been studied extensively as living arterial substitutes throughout the last 25 years, clinical translation is yet to come (Zhang et al., 2007a; Nemeno-Guanzon et al., 2012; Catto et al., 2014; Laurence et al., 2016). Strategies for vascular tissue engineering (vTE), and more specifically, for the fabrication of TEBV, differ from each other in terms of materials used, fabrication techniques, sources of selected cells and stimulation of the constructs towards tissue formation (Seifu et al., 2013; Wolf et al., 2016). Recent efforts have explored the potential of TEBV as *in vitro* cardiovascular models, aiming to bridge the gap between 2D cell cultures and *in vivo* models (Ryan et al., 2016). This approach not only improves our understanding of pathophysiology but also holds promise for advancing clinical therapies while reducing the need for animal testing (Robert et al., 2013; Wolf et al., 2016).

Two pivotal elements in the bioengineering of blood vessels or modeling the vascular wall are the scaffold and the vascular cells. The scaffold is a structure that should initially provide mechanical stability, sustain biological functions and exhibit biocompatibility and biodegradability. It is expected to support and stimulate the formation of three-dimensional (3D) tissue showing hierarchical structure (Ratner et al., 2004; Fu and Wang, 2018). On the other hand, vascular cells are expected to recapitulate the *orchestra* of physiological stimuli present *in vivo*, including mechanical. The exogenous collagen-matrix, in which vascular cells are included since the first steps of the biocasting process (González-Pérez et al., 2021), has recently shown to be able to stimulate endogenic production of extracellular matrix (ECM) from cells similarly to what happens in the native blood vessels (Ratner et al., 2004; Fu and Wang, 2018).

With respect to the development of the *ideal* scaffold, material selection plays an important role. Collagen, being one of the main components of the vascular ECM, is commonly used in vTE (Zhang et al., 2007b; Huang and Niklason, 2014), and more specifically, for the development of vascular wall models (Berglund et al., 2003; Wolf et al., 2009; Loy et al., 2017; Copes et al., 2019; Rachev and Shazly, 2019). The use of collagen is prompted by several favorable characteristics including weak antigenicity and robust biocompatibility along with promotion of cell adhesion, and biodegradability (Park et al., 2012; Sheehy et al., 2017; Copes et al., 2019). However, it falls short in terms of mechanical properties, particularly, their viscoelastic properties, narrowing their use in TE applications (Copes et al., 2019; Pien et al., 2021a). For vascular TE, this implies that the mechanical properties of cellularized collagen-based constructs are unable to withstand the high cyclic pressures, and the intrinsic elastic strains and stresses (Gaudet and Shreiber, 2012; Copes et al., 2019). Therefore, different research approaches were used to overcome this limitation, including 1) maintaining the construct’s structural integrity by chemical, physical or enzymatic crosslinking (Copes et al., 2019; Pien et al., 2021a), 2) blending with other natural biomaterials, e.g., fibronectin (Pezzoli et al., 2018) or elastin (Camasão et al., 2020), or 3) combining natural materials with synthetic biomaterials by developing multi-material scaffolds (Berglund et al., 2003; Sheehy et al., 2017; Copes et al., 2019). The latter includes blending before processing (Joy et al., 2020), co-extrusion or layer-by-layer processing methods (Wise et al., 2011), post-processing steps including dip coating (Huling et al., 2016), or using synthetic polymer scaffolds as a reinforcement for collagen-based models (Browning et al., 2012; Aguirre-Chagala et al., 2017).

A plethora of synthetic materials as such (i.e., without blending with natural materials) have already been investigated for vTE, with poly (ε-caprolactone) (PCL) (Ju et al., 2017; Pan et al., 2017; Wang et al., 2017; Wu et al., 2018; Pennings et al., 2019) merging as notable contender. The main advantages of synthetic polymers include their excellent reproducibility, mechanical properties’ tunability and control over shape, architecture and chemistry (Askari et al., 2017; Fortunato et al., 2017; Hielscher et al., 2018; Stowell and Wang, 2018). PCL is a semi-crystalline, bioresorbable polymer that is used for certain clinical applications and medical devices (Kade and Dalton, 2021), already approved by the U.S. Food and Drug Administration (FDA). In addition, PCL grafts have shown improved patency and endothelialization compared to commercially available non-degradable grafts such as expanded poly (tetrafluoroethylene) (ePTFE) (Mrówczyński et al., 2014).

The (reinforcement) scaffolds’ properties not only depend on the selected material, but are also strongly influenced by the architectural design of the matrix structure, which is mainly defined by the processing technique (Erdem et al., 2017; Miranda-Nieves and Chaikof, 2017). In turn, the architectural design affects prominently cell behavior in terms of adhesion, migration, proliferation and differentiation (Erdem et al., 2017; Miranda-Nieves and Chaikof, 2017). Therefore, the processing technique has an important influence on the resulting mechanical and biological properties of the developed (reinforcement) scaffold.

To process biomaterials into tubular constructs, multiple processing techniques have been proposed and studied. These processing techniques can be grouped into conventional and advanced techniques. Some examples of conventional techniques include gas foaming, moulding, solvent casting and dip coating (Dutta et al., 2017). More advanced techniques, include solution electrospinning (SES), three-dimensional (bio)printing (3D(B)P) and melt electrowriting (MEW) (Holland et al., 2018; Robinson et al., 2019; Jeong et al., 2020). Each one of them has its strengths and weaknesses, and will influence the resulting properties of the fabricated tubular construct (Pien et al., 2021b). SES and MEW are techniques that enable the production of nano- and micro-scale fibers, respectively, constituting an advantage with regard to mimicking the natural ECM in terms of hierarchical organization and properties (Bhardwaj and Kundu, 2010; Kade and Dalton, 2021). 3D(B)P allows control of material deposition down to the micron level (45–1600 µm) (Billiet et al., 2014; Murphy and Atala, 2014; Pedde et al., 2017). Both 3D(B)P and MEW offer the possibility to design complex geometries through computer aided design (Sears et al., 2016; Robinson et al., 2019). All three techniques present unique advantages to process materials serving tissue engineering applications, including vTE (Pien et al., 2021b).

An *in vitro* vascular wall model capable of recapitulating the cellular and mechanical environment of native vessels represents a valuable platform to study cellular interactions and signaling cascades, to test drugs and medical conditions under (patho)physiological conditions (Robert et al., 2013; Wolf et al., 2016). Through a previously developed cellularized collagen-based model, a proof of concept using vascular cells was realized to mimic the tri-layered native arterial structure, with the corresponding three vascular cell types (Loy et al., 2017). These collagen-based models (Bono et al., 2016; Loy et al., 2017; Camasão et al., 2018; Loy et al., 2018; Camasão et al., 2020; González-Pérez et al., 2021) showed promising results in mimicking the biological properties of a native vessel but lacked appropriate mechanical properties. More specifically, (non-reinforced) collagen-based models were unable to withstand the high pressures and stresses encountered in the blood vessels (Gaudet and Shreiber, 2012; Copes et al., 2019; Camasão et al., 2022).

The aim of this study was to improve the mechanical properties while maintaining the biological properties of this cellularized collagen-based model by reinforcing the model using a tubular polymeric reinforcement composed of PCL. As such, we aimed at recapitulating the mechanical properties of the wall of the vascular medium diameter vessel (3–5 mm). The effect of the processing technique (i.e., SES, 3DP and MEW) and the corresponding scaffold architecture were evaluated on the resulting mechanical and biological properties of the reinforced collagen-based model. As a reference, a fibroblast-cellularized collagen-based model without PCL reinforcement layer was used.

## 2 Materials and methods

### 2.1 Development of reinforcement scaffold using solution electrospinning

Solution electrospun reinforcement scaffolds were produced within the Polymer Chemistry and Biomaterials research group at Ghent University (Belgium). The in-house manufactured electrospinning (ES) set-up is composed of a high voltage source (Glassman High Voltage, Inc.; model series EL50P00, high voltage DC power), a motion controller (CWFW Ghent University), and a motor-driven syringe pumping system (New Era Pump Systems, Inc.; model Single Syringe Pump NE-300). The applied processing parameters were varied within the ES process (voltage 15–20 kV, flow rate 1.0–2.0 mL h^-1^ and needle-to-collector distance from 16–18 cm), after which an optimal set of parameters was selected (i.e., voltage of 18 kV, flow rate of 1.4 mL h^−1^ and needle-to-collector distance of 18 cm). ES was performed at 21°C and the relative humidity (i.e., ranging between 25% and 35%) was determined by a hygrometer which was present in the ES cabinet.

The homogeneous polymer solution (23.3 (w/v)% PCL (Medical grade PCL, Purasorb PC 12) in chloroform, stirred overnight; optimized concentration from the tested range between 16 and 25 (w/v)%) was transferred into a 20 mL syringe that was clamped into the syringe pumping system. The ES needle (inner diameter: 0.58 mm) was placed above the collector. A mandrel rotating around its axis (180 rpm, Inox stainless steel, 2 mm diameter) was applied during the process of ES to produce tubular constructs. For an easy release of the electrospun tubes from the mandrel, preheated mandrels (T = 80°C) were dip coated in molten poly (ethylene glycol) 8,000 g mol^-1^ (PEG8k) (T = 80°C). After performing ES, the mandrels were submerged in ddH_2_0 to dissolve the water-soluble PEG8k-coating (approx. 1 mm thickness) and allow an easy release of the developed tubular PCL constructs.

### 2.2 Development of reinforcement scaffold using melt electrowriting

Melt electrowritten reinforcement scaffolds were produced at the Department of Functional Materials in Medicine and Dentistry, Institute of Biofabrication and Functional Materials, University of Würzburg and KeyLab Polymers for Medicine of the Bavarian Polymer Institute (BPI), Würzburg. Tubular constructs of PCL (Medical grade PCL, Purasorb PC 12) were processed with a custom-made melt electrostatic writing device with a cylindrical and interchangeable collector (diameter of 3 mm). The motorization is based on an Aerotech axis system (PRO115) and uses the A3200 (Aerotech) software suite as coding and machine operating interface. A modified code has been developed similar to previous work (McColl et al., 2018) to move the collector in translational as well as rotational directions to allow precise fiber placement onto a steel mandrel in predetermined winding angles. For the extrusion of materials, polypropylene cartridges and 22G flat tipped needles (Nordson EFD) were used in all experiments. The printing temperature and pressure were set to 89°C and 0.65 bar, respectively (Jungst et al., 2019). Based on the dimension of the collagen-based model as previously described (Camasão et al., 2020) and based on earlier findings on evaluating MEW tubes for vascular TE (Jungst et al., 2019), the following predefined specifications were chosen for tubular construct generation: the length of the construct was set to 11.56 mm, the number of fiber layers on top of each other was set to 20, the angle at which the fibers are aligned in relation to the longitudinal axis (winding angle) was 70°, and the number of turning points (pivot points) of the construct was 8. For further description of the printing variables, we refer to previous work from McColl et al., 2018.

### 2.3 Development of reinforcement scaffold using three-dimensional printing

3D printed reinforcement scaffolds were produced at the Biomaterials, Biomechanics and Tissue Engineering group, Department of Materials Science and Engineering, Universitat Politècnica de Catalunya, Barcelona, Spain. 3D printed tubular PCL constructs were fabricated by a solvent-casting direct-write technique using a BCN 3D+ printer (BCN 3D technologies) as described previously (Chausse et al., 2021). In brief, the printer was modified to solvent cast inks through a syringe micro-nozzle with a 250 μm inner diameter (Nordson^®^). Moreover, the printer’s Y-axis was modified by introducing a carbon fiber rotating mandrel to print cylindrical structures.

PCL inks were prepared by dissolution of PCL pellets (Medical grade PCL, Purasorb PC 12) in chloroform (Sigma-Aldrich) at a 62.5% ratio (w/v) using a centrifuge (SpeedMixer™, AC 150.1 FVZ, FlackTek). The tubular shape was inspired by the Igaki-Tamai stent (Kyoto Medical Planning, Japan) design structure composed of rhombic cells and its dimensions were 3 mm in diameter and 20 mm in length with 10 peaks. The software Fusion 360™ (Autodesk) was used for the tubular construct design and the resulting Computer-Aided Design was exported to STL format. Finally, Slic3r (open source) was used to translate STL to G-code, which was needed for the 3D printer. PCL tubes were printed at 4 mm s^-1^ velocity.

### 2.4 Morphological characterization of the developed tubular constructs

Microstructural characterization of surfaces and cross-sections of the developed tubular constructs was conducted by Scanning Electron Microscopy (SEM) performed with a FEI Quanta250 SEM system (Thermo-Fisher) using a secondary electron detector. The SEM images were acquired with an acceleration voltage of 15 kV. Calculations of the fiber diameter and pore size were performed using ImageJ software.

### 2.5 Cells and cell culture

Neonatal human dermal fibroblasts (HDFs, C0045C, Gibco, Thermo Fisher Scientific) were cultured in an incubator at 37°C under constant supply of 5% CO_2_ in Dulbecco’s Modified Eagle Medium (DMEM, Gibco) supplemented with 10% fetal bovine serum (FBS, Gibco) and 1% Penicillin-Streptomycin solution (Pen-Strep, Gibco). Cells were cultured up to 90% confluency, then enzymatically detached and counted for sub-culturing or experimental use. For these experiments, cells at passage 7 were used.

### 2.6 Preparation of reinforced cellularized collagen-constructs

Type I collagen was extracted from rat tail tendons, solubilized in 0.02 N acetic acid at a concentration of 4 g L^−1^, sterilized and processed according to a previously reported protocol (Rajan et al., 2007). The collagen solution was mixed with a neutralizing buffer solution (3.5× DMEM supplemented with 10 mM HEPES and 60 mM NaOH) and a suspension of HDFs in culture medium (DMEM supplemented with 10% of FBS and 1% of Pen-Strep) at 4°C in a ratio of 2:1:1, respectively. The final collagen concentration in the gel was 2 g L^−1^ (pH 7.2) and the cell density was 1.5 × 10^6^ cells∙ml^-1^. The final solution was poured in a 48-well custom-made plate containing a central PEEK mandrel (Ø = 2.985 mm), in 4 different conditions: (1) no reinforcement (COL, reference), (2) a solution electrospun (SES), (3) a three-dimensionally printed (3DP) or (4) a melt electrowritten (MEW) reinforcement (i.e., tubular reinforcement construct placed around the mandrel before adding the final solution). The tubular gel was gently detached from the wall and medium was added to fill the well. The plate was incubated at 37°C and 5% CO_2_ for 3 and 7 days. Culture medium was changed every day. Four samples for each condition and time point were prepared.

### 2.7 Mechanical characterization of tubular constructs

(a) Evaluation of gel compaction

The length and the outer diameter of the developed constructs (gel and reinforcement layer) were measured to evaluate gel compaction after 3 and 7 days of culture. A caliper was used for measuring the length while a scanning laser interferometer (LaserMike 136, Series 183B, NDC Technologies) was applied to determine the external diameter. The inner diameter was known and equal to the diameter of the central PEEK mandrel (Ø = 2.985 mm), which enabled volume calculation of each sample. Length and wall thickness were calculated for each sample at both time points. Data are expressed as mean ± standard error of mean (n = 3).

(b) Evaluation of visco-elastic properties

The viscoelastic properties were evaluated by tensile stress relaxation tests using an Instron E1000 (Instron Corporation) equipped with a 5 N load cell. Ring-shaped samples (length approx. 4 mm) were placed on *ad hoc* made L-shape grips and tested in a phosphate-buffered saline (PBS) bath at 37°C to mimic physiological conditions. A pre-strain of 5% was applied to the samples followed by 5 progressive stress relaxation cycles each consisting of 10% strain ramps (5%∙s^−1^ strain rate) and 10 min of relaxation. After the 5th cycle (55% of deformation), the test continued with the same strain rate up to sample failure. The measured load was divided by the initial cross-sectional area of the sample to obtain engineering stresses. Strain was determined as the variation of the distance between the grips (l-l_0_) divided by the initial distance (l_0_), the point of zero force calculated with the sample’s original circumference. E_E_ is defined as the equilibrium elastic modulus, and E_0_ as the initial (instantaneous) elastic modulus. Data are expressed as mean ± standard error of mean (n = 3).

### 2.8 Biological characterization of tubular constructs

(a) Evaluation of cell viability (Resazurin)

AlamarBlue Cell Viability assay (Thermo Fisher Scientific) was performed according to the manufacturer’s instructions. Briefly, the culture medium was removed from each well and replaced by 850 µL of resazurin solution in DMEM (1X) at each time point (i.e., day 3 and 7). The plate was then incubated for 4 h at 37°C and in 5% CO_2_ to allow the reduction of resazurin into the pink and highly fluorescent resorufin. Aliquots (100 μL, n = 4) from each sample (n = 3) were transferred to a 96-well plate and fluorescence was measured (λ_ex_ = 560 nm; λ_em_ = 590 nm) in a multi-well plate spectrophotometer (SpectraMax i3x, Molecular Devices). Results were normalized over the non-reinforced collagen-based model (COL) at day 3. Data are expressed as mean ± standard error of mean (n = 4).

(b) Immunofluorescence staining (Phalloidin for actin/DAPI) and histochemistry

After 3 and 7 days of maturation, the constructs were fixed with 3.7% formaldehyde (Sigma) for 60 min, washed with PBS 1X (1 × 20 min, 2 × 2 min), and treated with 0.5% Triton X-100 in PBS1X for 5 min at room temperature (r.t.) to permeabilize the cells. Subsequently, the constructs were rinsed two times with PBS 1X. Afterwards, the samples were blocked in a 3% bovine serum albumin (BSA) in PBS solution for 20 min at r.t. After removing the blocking solution, the samples were incubated at r.t. for 2 h with Collagen I primary antibody (mouse) (1:1000, Novus Biologicals) in blocking solution. The samples were then rinsed with PBS 1X and incubated for 1 h with a goat anti-mouse Alexa Fluor 488 secondary antibody (1:200, Life Technologies) and with Rhodamine-conjugated phalloidin (1:200, Sigma), both prepared in blocking solution. After rinsing the samples with PBS 1X, 4′,6-diamidino-2-phenylindole (DAPI, 1:3000, Thermo Fisher Scientific) was used to stain cell nuclei. Images were obtained using an LSM 700 confocal laser scanning microscope (Zeiss) controlled by ZEN 2009 software for image acquisition and further analysis.

Histochemistry (HC) was performed to observe collagen, cell distribution and compaction. Tubular samples were rinsed in PBS and fixed in 3.7% formaldehyde (Sigma-Aldrich) for 60 min. Fixed samples were then embedded in paraffin and cut into circumferential cross-sections of 5 μm. Sections were deparaffinized with toluene, rehydrated with successive washes with ethanol in deionized water (dH_2_O) at decreasing concentrations (100%, 95%, 80%, and 0%), refixed in Bouin solution overnight and stained with a modified Masson’s trichrome procedure. The following dye solutions were added to stain the nucleus, the cytoplasm and collagen: Weigert’s iron hematoxylin, acid fuchsin with xylidine ponceau and light green SF yellowish, respectively. Images were obtained by an Olympus BX51 microscope (Olympus Canada Inc.).

(c) Evaluation of gene expression and ECM deposition

The expression of different target genes by HDFs was assessed by qRT-PCR. RNA was isolated from each sample using TRIzol^®^ Reagent (Thermo Fischer Scientific), according to the supplier’s instructions. Briefly, the constructs were immersed in TRIzol^®^ reagent and homogenized with tissue grinders. Chloroform was added to separate the organic layer from the aqueous phase containing RNA followed by isopropanol for its precipitation. The RNA pellet obtained after centrifugation was washed with 75% ethanol, resuspended in RNase-free dH_2_O and stored at −70°C. The RNA content and purity were determined with Nanodrop (ND-1000 Spectrophotometer, NanoDrop Technologies, Inc., Wilmington, DE, United States). High purity levels were achieved (A260/280 > 1.8). The QuantiTect^®^ Reverse Transcription Kit (Qiagen Inc., Toronto, ON, Canada) was used to reverse transcribe the isolated RNA into cDNA using a thermal cycler (PTC-200, MJ Research), according to the manufacturer’s guidelines. The real-time PCR was performed using a 7500 Fast Real-Time PCR System (Applied Biosystems, Thermo Fisher Scientific). TaqMan^®^ Gene Expression Assays targeting GAPDH (Hs03929097_g1), elastin (Hs00355783_m1), fibrillin-1 (FBN1, Hs00171191_m1) and Ki-67 (MKI67, Hs01032443_m1) with TAQMAN universal Master mix II with UNG (all purchased from Applied Biosystems) were employed in duplicate for each sample. Finally, the relative quantification of mRNA levels (fold change in relation to the control gene GAPDH and to the condition of the collagen-based model at day 3) were calculated using the 2^−ΔΔCT^ method (n = 3).

### 2.9 Statistical analysis

Data was analyzed using R Studio (Version 1.3.1093, RStudio, PBC, Boston, MA, United States), but due to the limited sample size (n = 3), data was transformed to their ranks. The effect of the condition (control vs. SES vs. 3DP vs. MEW), the effect of time (day 3 vs. day 7) and their interaction was analyzed (two-way ANOVA test on ranked values). Subsequently, significant effects were further investigated using the package “multcomp” for multiple pairwise-comparisons of the main effects (condition & time) and the package “emmeans” to investigate interactions (condition*time). The symbols representing the different significant levels are indicated on the graphs, and/or defined in the captions (i.e., ns = p > 0.05; * = p ≤ 0.05; ** = p ≤ 0.01; *** = p ≤ 0.001).

## 3 Results and discussion

Tubular polymeric scaffolds were fabricated in PCL by 1) solution electrospinning (SES), 2) 3D printing (3DP) and 3) melt electrowriting (MEW). These PCL tubes were then used as reinforcement for the previously developed collagen-based model (Camasão et al., 2018; Loy et al., 2018; Camasão et al., 2020). The non-reinforced collagen-based model was used as a reference throughout this research work, and this manuscript. The effect of the processing technique (i.e., SES, 3DP and MEW) and the corresponding scaffold architecture, on the resulting mechanical and biological properties of the reinforced collagen-based model were evaluated.

### 3.1 Morphological analyses

**FIGURE 1:**
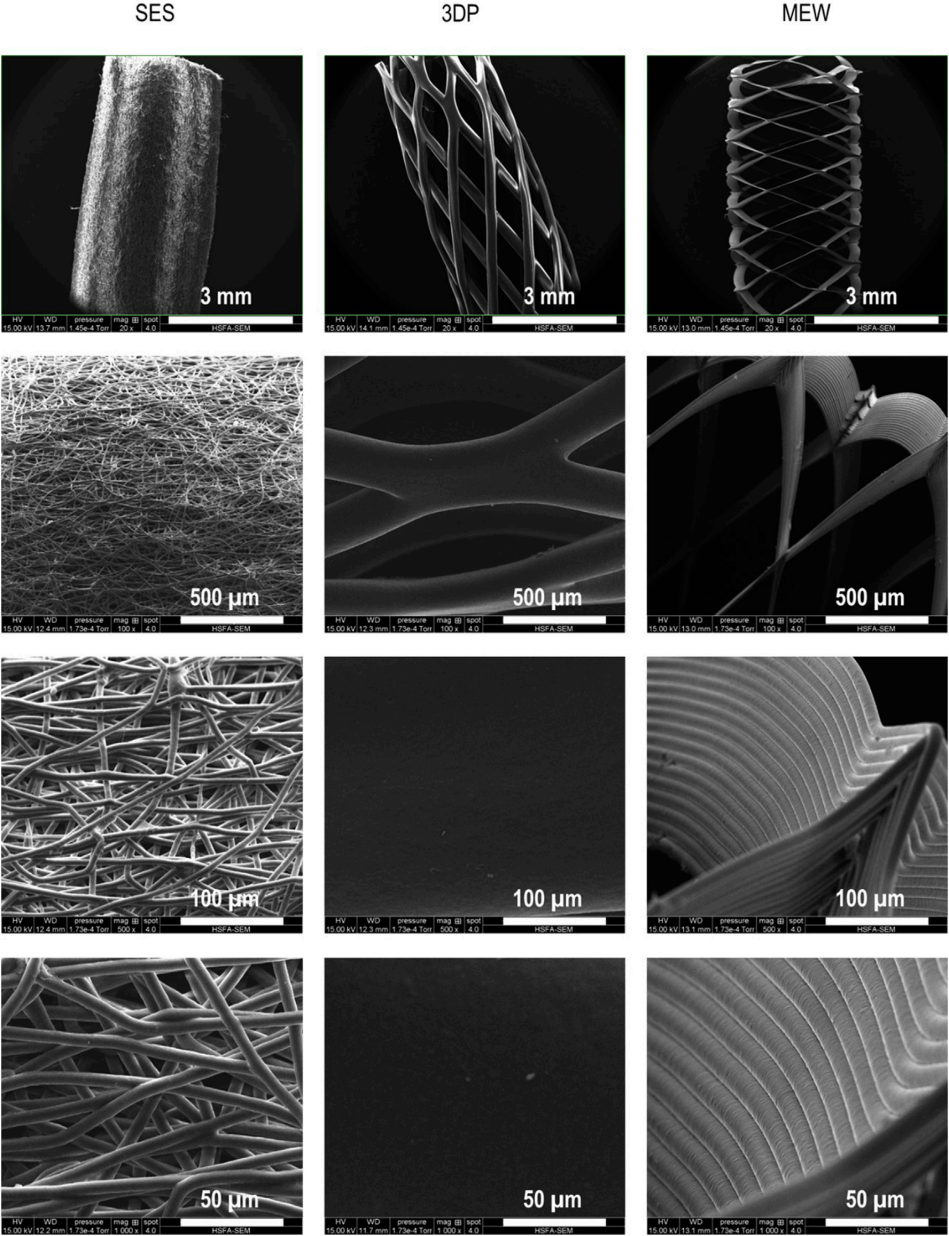
Visualization of the reinforcement tubes using SEM imaging, processed by SES, 3DP and MEW. Fiber diameters of SES, 3DP and MEW amounted 6.58 ± 0.30 µm, 237.04 ± 12.51 µm and 13.16 ± 0.67 µm, respectively.

As a first step in the evaluation of the effect of the selected processing technique on the scaffold’s architecture, SEM was performed to visualize the fibers’ alignment and diameter. Figure 1 shows the fibers of the developed scaffolds using SES, 3DP and MEW at different magnifications. The SES tubes showed a randomly oriented fiber distribution, closely resembling the native ECM. SES scaffolds (fabricated on rotating mandrels at low rpm) are known for such fiber arrangements, representing one of the main advantages of this processing technique (Jiang et al., 2014). The average diameter of the SES fibers was 6.58 ± 0.30 µm, the smallest of the three techniques compared in this paper. The largest fiber diameters were measured in the 3DP structure, with an average fiber diameter of 237.04 ± 12.51 µm. The visualization of the 3DP structure also confirmed the predefined design and controlled deposition of the fibers down to 100 µm level. MEW is known as a technique that allows the deposition of micrometer (up to ±10 µm) scale fibers in a predefined design (Pien et al., 2022). This was confirmed by SEM images, which indicated that the average fiber diameter of MEW constructs was 13.16 ± 0.67 µm. The multiple layers of the MEW fibers were perfectly deposited onto each other, leading to a precisely defined scaffold architecture.

Next, the SEM images were analyzed to assess the pore size of the different tubular scaffolds. The tightly packed fibers of the SES tubes showed the smallest pores, with widths (i.e., shortest distance between 2 struts) ranging from a minimum of 7 ± 2 μm up to a maximum of 32 ± 10 μm. Conversely, the 3DP tubes showed larger pore sizes, being 619 ± 58 μm in width and 1800 ± 13 μm in length (i.e., greatest distance between 2 struts). Lastly, the MEW tubes’ pore size was 698 ± 50 μm in width and 1803 ± 99 μm in length (see Figure 1). The different PCL tubular scaffolds were also characterized in terms of their thickness. The 3D printed tubes, consisting of only a single fiber monolayer, exhibited a thickness of 216.43 ± 49.27 µm. This value was similar to the average fiber diameter shown in the SEM images (i.e. 237.04 ± 12.51 µm), confirming that the scaffold’s structure is based on one single strut. For SES and MEW, a thickness of 613.07 ± 138.01 µm and 191.7 ± 5.50 µm was obtained, respectively. The small standard deviation obtained for the MEW tube, compared to SES, also evidences the excellent reproducibility and precision regarding fiber deposition of the MEW technique.

All developed tubular scaffolds were fabricated with an inner diameter of 3 mm, aiming at small-diameter (i.e., < 6 mm) vTE (Wang et al., 2020). PCL as a biocompatible, FDA-approved and overall easily processable via numerous processing methods was chosen as the common ground for analysis of the different fabrication techniques. While it features a fatigue behavior when extended above the elastic region of stress and is not very elastic in bulk, processing it with specified geometries into fibrous constructs can mitigate some of these shortcomings (de Valence et al., 2012). Issues, including calcifications, arising after implantation of PCL scaffolds have been reported (de Valence et al., 2012). Nevertheless, the biodegradability of PCL proves to be a major benefit, especially regarding tissue regeneration and recent research efforts have been focused on tuning this behavior with different construct geometries and topographies (de Valence et al., 2012; Wang et al., 2020; Dias et al., 2022).

It is hypothesized that differences in fiber alignment, diameter, pore size and hence scaffold architecture, will influence the collagen compaction, and consequently, both mechanical and biological properties. These properties will be described in the upcoming sections.

### 3.2 Mechanical characterization

(a) Evaluation of gel compaction

**FIGURE 2:**
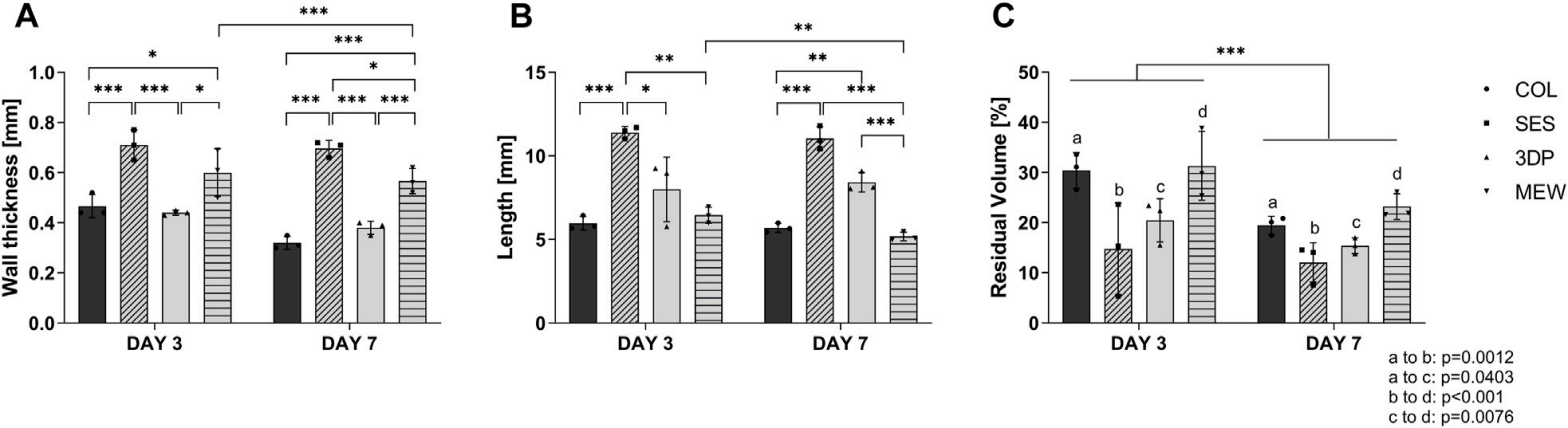
Compaction of the cellularized tubular gels without reinforcement (COL), and with the different types of reinforcement (SES, 3DP and MEW) at day 3 and day 7 of maturation. **(A)** Wall thickness of the tubular gel. **(B)** Length of the collagen gel. **(C)** Residual volume of the collagen gel following compaction, normalized against their initial volume. (* = p ≤ 0.05; ** = p ≤ 0.01; *** = p ≤ 0.001).

Upon visual inspection, differences in gel compaction (both in length and thickness) were already observed at day 3 and day 7 when comparing the tubular gels reinforced by the three different processing techniques (Figure 2). In case of a SES reinforcement layer, the collagen gel compacted as an outer layer around the SES scaffold. For the 3DP scaffold, the collagen gel compacted thereby filling the large holes of the tubular structure. After maturation during 3 and 7 days, the collagen gels of COL, and MEW were visually very similar in thickness and length. Seemingly, the HDFs-mediated collagen compaction was not influenced by the presence of the MEW scaffold. This is probably due to the predefined architecture resulting from nicely stacked, thin PCL fibers that lead to a flexible structure. The flexible structure is anticipated to enable the deformation of the MEW tube, and therefore, the MEW scaffold shrunk in length upon compaction of the collagen gel (to the same length and thickness as the COL condition). When comparing COL to 3DP and SES, the cellularized collagen gel was more spread out in length over the 3DP and SES tube and was thinner in wall thickness.

The differences in scaffold architecture along with flexibility of the developed tubular reinforcements has a major impact on collagen gel compaction. The SES tube exhibited a high surface area-to-volume ratio, yet small pores and was less deformable compared to the MEW construct, but more deformable compared to the 3DP tube (*vide infra,* 3.2.b). The SES construct features randomly aligned fibers, which are likely to distribute luminal pressure in an isotropic manner across the construct. Blood vessels are structured in a highly anisotropic fashion, distributing the luminal pressure in a different way as a pure SES construct would do. To emulate this, an anisotropic fiber alignment will be necessary to recapitulate this behavior. Both 3DP and MEW scaffolds feature a rhombus geometry of fibers, which is inspired on the anisotropic orientation of most ECM fiber components usually found in blood vessels (Holzapfel and Weizsäcker, 1998; Holzapfel et al., 2000). This shape has also been consistently used in many publications to better recapitulate the J-shape stress-strain behavior of blood vessels (van Genderen et al., 2021; McCosker et al., 2022; Federici et al., 2023). In general, many different mechanical characteristics can be recapitulated by fibrous scaffolds through adopting their geometry (Olvera et al., 2020; McCosker et al., 2022; Cao et al., 2023) rendering them applicable to many potential medical areas, amongst which bone and cartilage regeneration or vessel replacement (Daghrery et al., 2023). An interesting study from Pickering et al., 2022 evaluated how geometric properties can be exploited to tailor the mechanical properties of tubular scaffolds.

**FIGURE 3:**
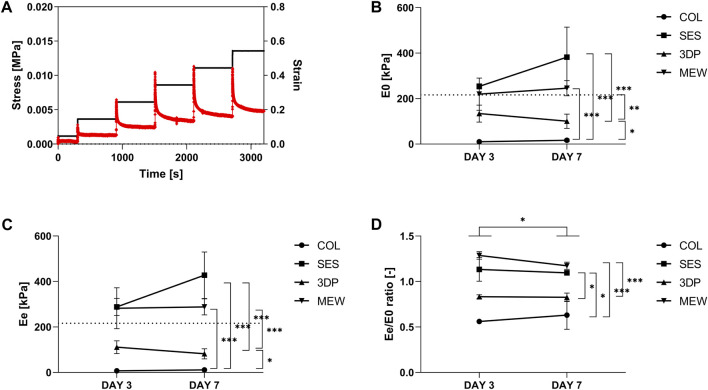
Mechanical evaluation of fibroblast-cellularized COL (reference), SES, 3DP and MEW reinforced collagen-based models (day 3 and day 7 of maturation). **(A)** Example of a stress-strain curve, obtained from stress-relaxation testing using Instron. Example represents a COL sample on day 3. **(B)** E_0_, initial (instantaneous) elastic modulus. **(C)** E_e_, equilibrium elastic modulus. **(D)** Ratio of E_e_/E_0_. The dashed line in **(B)** and **(C)** corresponds to an incremental elastic modulus of 216 kPa, as an example calculated for the abdominal aorta of a human aged 27 years old (diameter of 13.2 mm and thickness of 1.59 mm). (* = p ≤ 0.05; ** = p ≤ 0.01; *** = p ≤ 0.001).

Depending on the fabrication method exploited, the mechanical response of the construct was altered. The tested SES samples showed a rather stiff mechanical response due to the tightly packed fibers that are generated during the electrospinning process, whereas the 3DP and MEW constructs featured larger voids in between the fibers that allow for a certain level of elastic deformability (Figures 3B–D). This deformation is also related to the varying width of the collagen gels on the different construct types. Comparing the total width of the gel reveals a clear difference between SES and 3DP samples as compared to the pure COL gel and MEW-reinforced gel (Figures 2A,B). This is also observed when comparing the overall residual collagen gel volumes of the respective constructs after day 3 (SES = 14.78 ± 9.14 mm³; 3DP = 20.47 ± 4.30 mm³; COL = 30.45 ± 3.57 mm³; MEW = 31.33 ± 6.86 mm³) and day 7 (SES = 12.06 ± 3.91 mm³; 3DP = 15.39 ± 1.58 mm³; COL = 19.47 ± 1.81 mm³; MEW = 23.22 ± 2.55 mm³) respectively (Figure 2C). This contractile behavior of collagen gels has been documented and modeled before and is induced by cell remodeling of the gels as well as the geometry and surrounding of the gel (Ohsumi et al., 2008; Aghvami et al., 2013). While the COL gel alone and MEW samples allow the gel to freely compact itself isometrically due to absence of, or very little resistance from the construct, the overall reduction in volume is less than the one measured for the SES scaffold or the 3DP construct. The latter two constructs restrict the gel in one or multiple directions, forcing it to compensate for the overall contraction to occur in the remaining unobstructed (vertical) directions, resulting in a higher overall compaction (Figure 2).

Even though both 3DP and MEW result in constructs with high porosity, the mechanical behavior of the two sample types is not identical. A distinct difference between the 3DP and MEW fibers encompasses the overall geometrical morphology, where one solid fiber (d = 237.04 ± 12.51 µm) makes up the main part of the geometry in case of 3DP in contrast with a stack of thin fibers (d = 13.16 ± 0.67 µm) in case of the MEW construct.

(b) Evaluation of visco-elastic properties

As shown by SEM imaging, the selected processing technique affects the developed scaffold’s architecture on both the micro- and the macro level, thereby influencing the resulting mechanical properties. The effect of the reinforcement scaffold on the viscoelastic properties of the collagen-based model was evaluated by stress-relaxation testing (Supplementary Figure S2). An example of the stress response of a collagen gel (COL, day 3) after five cycles of 10% deformation followed by 10 min relaxation is shown in Figure 3A. The initial elastic modulus (E_0_, Figure 3B) corresponds to the data obtained immediately after the deformation, while the equilibrium elastic modulus (E_e_, Figure 3C) corresponds to data obtained just before a new deformation (i.e., at the end of the 10 min deformation) cycle was initiated. Figure 3D represents the ratio between E_e_ and E_0_ and provides information on the predominance of the viscous or the elastic component. A higher ratio E_e_/E_0_ implies that the viscous component becomes less representative, and that the elastic behavior becomes predominant (ratio >1: elastic behavior predominates; ratio <1: viscoelasticity predominates) (Camasão et al., 2020).

The incorporation of a reinforcement scaffold (i.e., SES, 3DP or MEW) in the cellularized collagen-based model (COL) resulted in a significant increase in both E_0_ and E_e_ (*p* < 0.05). On day 3, the SES, 3DP and MEW reinforcement led to an equilibrium elastic modulus of 253.66 ± 11.63 kPa, 134.06 ± 37.31 kPa and 219.54 ± 70.76 kPa, respectively, compared to 10.41 ± 7.89 kPa for COL. This implies that the E_0_ of SES and MEW is quite similar at day 3. However, at day 7, SES showed a higher E_0_ than the MEW reinforcement, albeit not statistically significant. The MEW condition also showed significant differences compared to COL and 3DP conditions (*p* < 0.05). A similar trend was observed for E_e_ (Figure 3C). On day 7, the developed collagen gels showed stresses (for strains between 20% and 55%) in the range of [5–15] kPa for COL, [80–350] kPa for SES, [20–70] kPa for 3DP and [100–190] kPa for MEW.

When considering the ratio of E_e_ and E_0_ (at day 3), the highest ratio was observed for MEW (1.29 ± 0.04), followed by SES (1.13 ± 0.13), 3DP (0.83 ± 0.02) and COL (0.85 ± 0.41). There is a significant difference in between both timepoints (day 3 and day 7), with *p* = 0.0364. In between the conditions, a significantly higher ratio E_e_/E_0_ was observed for SES (*p* = 0.0366) and MEW (*p* < 0.001) compared to the reference COL. The MEW condition was also significantly different from the 3DP condition (*p* < 0.001). The predefined architecture of the MEW reinforcement scaffold, including the angle at which the fibers were aligned in relation to the longitudinal axis (winding angle of 70°) and the number of turning points of the scaffold (8 pivot points), enabled a flexibility and elasticity that neither the 3DP nor the SES tube showed. This already resulted in differences in gel compaction when comparing the MEW reinforced collagen gel with the 3DP and SES reinforced gels (Section 3.2.a), and was here confirmed by the high E_e_/E_0_ ratio, implying a predominantly elastic behavior.

These data highlight that, apart from material selection, the selected processing technique has an important influence on the resulting mechanical properties of the developed tubular scaffold. The randomly deposited SES fibers showed the best mimicry compared to the native ECM, and also resulted in a significant increase of the elastic modulus compared to COL (reference). The collagen gel spread out over the full length of the SES reinforcement tube. This implies that the less deformable structure of the randomly oriented SES fibers did not enable a change in the reinforcement tube’s architecture (such as reduction in length) upon collagen compaction. A similar observation was made for the 3DP reinforcement scaffold. Conversely, the MEW tube also showed a significant increase in elastic modulus compared to COL, but still enabled the HFDs to compact the collagen as they would do without the presence of a reinforcement scaffold (by “shrinking” upon collagen compaction). This is also due to the predominance of the elastic behavior in the MEW reinforcement tube, as was also shown by the E_e_/E_0_ ratio (Figure 3D).

In literature, collagen-based models have already been described, referring to elastic moduli of maximum 30 kPa for a (non-reinforced) collagen-based model (Meghezi et al., 2012; Seifu et al., 2018; Camasão et al., 2020). Different approaches have been evaluated in an attempt to improve the mechanical properties (i.e., increase elastic modulus) of collagen-based models, including increasing cell density (Camasão et al., 2018) or through addition of elastin-like recombinamers (Camasão et al., 2020) have reported a significant increase between initial and equilibrium elastic moduli of 40% and 50%, respectively, for their developed elastin/collagen-based model (30% elastin) compared to the collagen-based model (used as a benchmark in the present study). However, the initial elastic modulus E_0_ did not exceed 30 *versus* 50 kPa after 3 and 7 days, respectively, and the equilibrium elastic moduli E_e_ did not exceed 20 kPa after 7 days for any of the tested conditions. Compared to these results, the polymeric tubular reinforcements used herein greatly improved the mechanical properties of the collagen-based model with an E_0_ of 382.05 ± 129.79 kPa, 100.59 ± 31.15 kPa and 245.78 ± 33.54 kPa, respectively for SES, 3DP and MEW at day 7 of maturation, respectively.

In the work of “Mc Donald’s Blood Flow in Arteries,” the reported values on the pressure-strain elastic (Peterson) modulus (E_p_) of human arteries range between [0.52–1.18]∙10^6^ dyn cm^-2^ or [52–118] kPa, depending on the type of artery and the age of the patient (Nichols et al., 2011). This can be translated into an incremental (∼Young’s) elastic modulus by taking into account the diameter and the thickness of the blood vessel. As an example, for the abdominal aorta of a human aged 27,years old (diameter of 13.2 mm and thickness of 1.59 mm) (Nichols et al., 2011), an E_p_ of 52 kPa corresponds to an incremental elastic modulus of 216 kPa (O’Rourke et al., 2002; Dijk et al., 2005). For this example, the MEW construct with 245.78 ± 33.54 kPa is approximating this value the closest of all investigated constructs.

In conclusion, the tubular, polymeric reinforcement scaffolds significantly improved the mechanical properties of the collagen-based model, reaching values close to the mechanical properties of native arteries (Van Andel et al., 2003). The three processing techniques have also resulted in different scaffolds’ architectures, which can be linked to differences in the mechanical properties.

### 3.3 Biological performances

(a) Evaluation of cell viability

**FIGURE 4:**
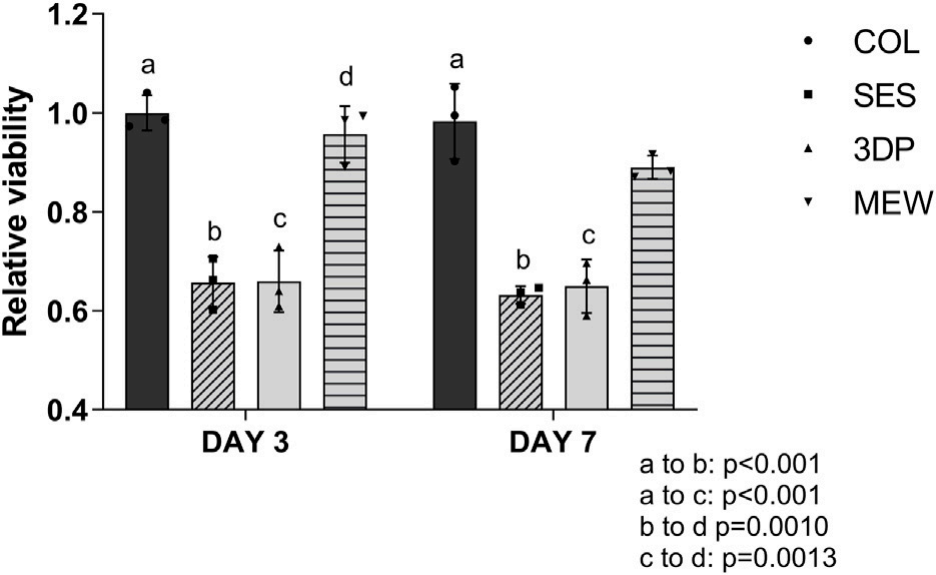
Relative viability based on a Resazurin assay at day 3 and day 7 on COL (reference), and on the reinforced collagen-based model using a SES, 3DP and MEW scaffold.

HDFs’ metabolic activity was evaluated between groups at 3 and 7 days and the results were normalized towards the day 3 COL condition (as the non-reinforced collagen gel acts as reference in this study). The results (Figure 4) showed a significant decrease in cell metabolic function between the SES and 3DP condition, compared to COL and MEW. This was observed both on day 3 and day 7. However, no significant differences were observed between the COL control and the MEW condition, suggesting that cell viability was not affected by the presence of the MEW scaffold.

The lower metabolic activity observed in the SES and 3DP reinforced model, compared to COL and MEW, can be linked to the differences in materials’ architecture (Section 3.1) and mechanical properties (Section 3.2). The architecture of polymeric scaffolds, and more specifically, the scaffold properties such as fiber diameter and pore size, can greatly impact cell infiltration, - attachment, - proliferation and - differentiation (Pham et al., 2006). Soliman et al., 2011 demonstrated how tightly packed fibers in SES scaffolds gave rise to small pores ranging from 7 to 32 µm (Section 3.1), which have shown to decrease cell metabolic activity when compared to electrospun scaffolds with a lower fiber density and larger pore size (i.e. 44–64 μm) (Soliman et al., 2011). 3DP scaffolds showed larger pore sizes (619 ± 58 µm in width; 1800 ± 13 µm in length, Section 3.1), which also might negatively affect fibroblasts’ metabolic activity. It has already been suggested that fibroblasts exhibit a superior cell attachment and growth on scaffolds presenting pore sizes smaller than 160–200 µm (Bružauskaitė et al., 2016). The deformability of the MEW scaffold combined with the collagen gel and cells was mainly observed in the longitudinal direction (i.e., length of the tubular reinforcement, see also Figure 2), thereby changing the architecture and decreasing the pore size. Furthermore, studies on the surface morphology have shown that micro-topography affects cell metabolism in several cellular types (Von Recum and Van Kooten, 1996; Mobini et al., 2021). Fibrous topographies influence cell proliferation and tissue formation, which in turn promotes better metabolic activity through orientation and direction (Alshomer et al., 2017). While collagen and MEW are effective at inducing directionality and promoting cell orientation, 3DP does not show the same effect due to the large fiber diameter. Randomly spun SES has already been reported not to induce directionality without further guiding queues (Jungst et al., 2019). These findings do explain the behavior recorded in the presented graph and the decrease in metabolic activity in the 3DP and SES reinforced models compared to collagen gels and the MEW reinforced model. In addition, simulations of collagen gel compaction showed an increase in stress levels within the gel upon compaction (Ohsumi et al., 2008; Aghvami et al., 2013). This effect might also be responsible for the reduced cell viability in the compacted gels of SES and 3DP gels while the elasticity of MEW constructs allows for uniform compaction with much lower overall stress exerted on the cells.

(b) Evaluation by immunofluorescent staining and histochemistry

**FIGURE 5:**
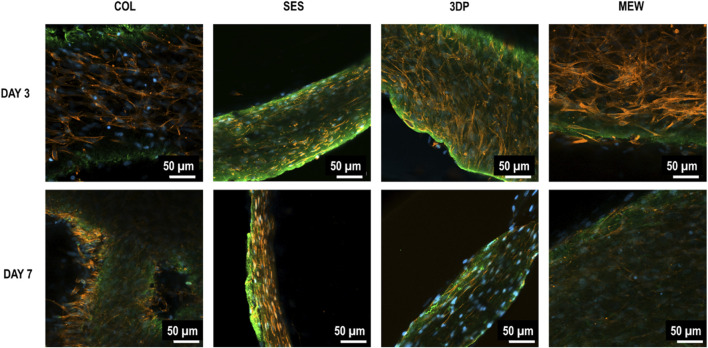
Immunofluorescence stained 2D images of fibroblast-celullarized tubular collagen-based gels, without (COL, reference) and with reinforcement (SES, 3DP and MEW) at day 3 and day 7 of maturation: Collagen I (green), F-actin (red), and cell nuclei (blue).

The samples’ cross-sections were stained to evaluate collagen thickness, cell distribution and alignment in the four different conditions at day 3 and day 7 via immunofluorescence and histochemistry. Figure 5 shows immunofluorescence staining of collagen (green), F-actin (red) and cell nuclei (blue), highlighting the differences in cell morphology between the conditions.

**FIGURE 6:**
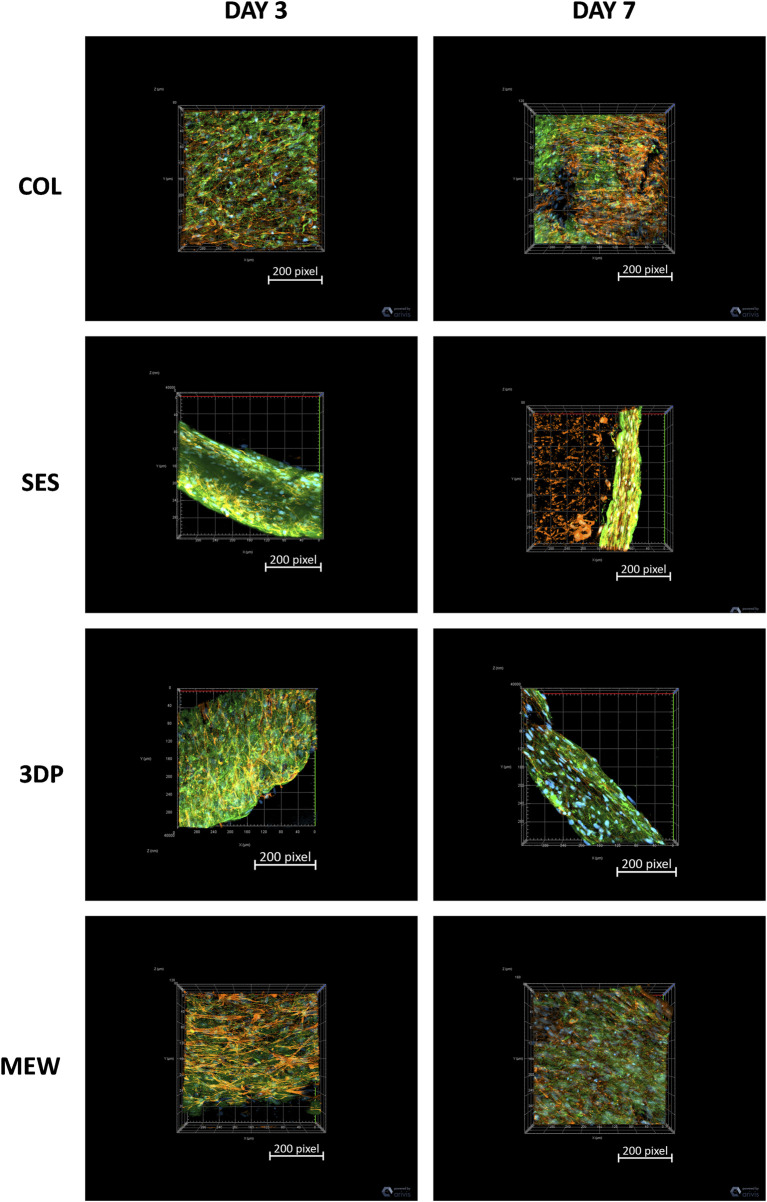
Immunofluorescence stained 3D images of fibroblast-celullarized tubular collagen-based gels, without (COL, reference) and with reinforcement (SES, 3DP and MEW) at day 3 and day 7 of maturation: Collagen I (green), F-actin (red), and cell nuclei (blue).

The 2D images of COL and MEW reinforced gels showed a thicker cross-section of the non-reinforced collagen gel (reference, COL), with HDFs being uniformly distributed within the gel. The SES and 3DP conditions showed thinner collagen gels with fewer total cells in comparison with MEW and COL conditions. The 3D images (Figure 6) of COL and MEW reinforced gels showed that cells were nicely distributed within the gel, forming a cellular network, with no visual differences between day 3 and day 7. Moreover, no particular cell alignment was displayed. The same was observed at day 3 in the 3DP samples. However, at day 7, the 3DP samples showed no cell network formation. The SES condition did not show a cellular network at any time point. Differences were also observed in cell morphology, as HDFs in the COL and MEW conditions showed elongated actin, compared to the smaller and less extended actin cytoskeleton of HDFs in the SES and 3DP conditions.

**FIGURE 7:**
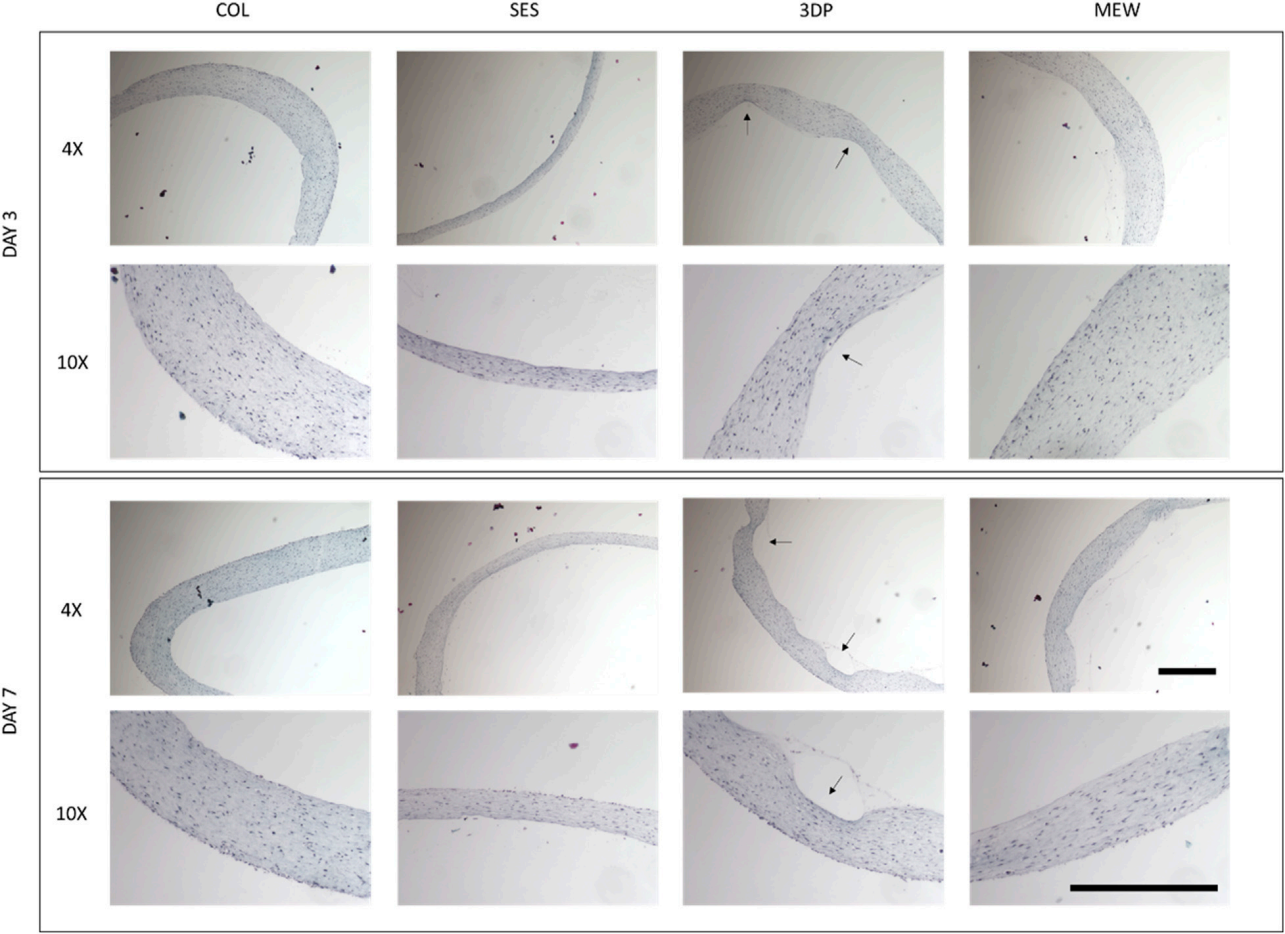
Histological analysis: Masson Trichrome staining of the fibroblast-cellularized tubular collagen-based gels, without (COL, reference) and with reinforcement (SES, 3DP and MEW) at day 3 and day 7 of maturation. The black arrows indicate the original location of the 3D printed scaffold. Scale bars represent 500 μm at ×4 and ×10 magnification.

Histological images (Figure 7) showed the cellularized collagen gel, its compaction and the cell distribution at days 3 and 7, while the reinforcement is not shown in these histological images. The COL condition showed a thick gel at day 3, with a decrease in thickness by day 7, confirming the observations described in Section 3.2a regarding gel compaction. For the SES condition, a very thin collagen gel was observed, lining the SES tube as an outer layer. The 3DP sample displayed the collagen gel filling the pores of the reinforcement scaffold’s structure, surrounding the one single thick fiber (as indicated by the black arrows in Figure 7). A thicker collagen gel was observed in the MEW condition, filling the structure completely.

Using both imaging techniques, it is clear that the COL and the MEW conditions are very similar. Both showed a thick and highly cellularized collagen gel, supporting the hypothesis that nor the collagen gel’s compaction nor the biological activity were impacted by the presence of the MEW reinforcement scaffold (Section 3.2a and 3.3a). In the SES condition, the small pores and dense structure (Section 3.1) did not allow the collagen and cells to penetrate, which resulted in a sharp contrast between the reinforcement scaffold and the collagen gel. The 3DP scaffold turned out to be even less deformable (Section 3.1 and 3.2b), and its large pores were filled with collagen gel, which resulted in a slender construct. Consequently, both SES and 3DP conditions showed thinner collagen gels, spread out in length throughout the scaffold. This difference in collagen gel thickness might be due to the architecture and lower deformability of these tubes, dictated by the different processing techniques. Furthermore, we hypothesize that the thinner gels in SES and 3DP conditions resulted in a decreased cell viability when compared to the COL and MEW conditions (as described in Section 3.3a).

In conclusion, it is clear that the different processing techniques and tubes’ architecture influenced cell behavior, in terms of metabolic activity and attachment, as well as collagen gel compaction. Moreover, differences were also observed in the cells’ morphology when comparing the conditions, varying from a dense network of randomly dispersed fibroblasts, with elongated actin cytoskeleton, within the thicker collagen gel of COL and MEW samples, to fibroblasts presenting smaller cytoskeletons, which were compressed together within the thinner collagen gel for the SES and 3DP conditions.

(c) Evaluation of gene expression and ECM deposition

**FIGURE 8:**
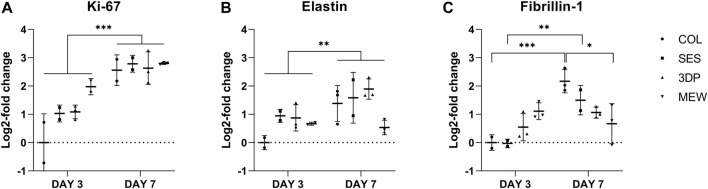
Gene expression of **(A)** Ki-67, **(B)** elastin and **(C)** fibrillin-1 and Ki-67 in the non-reinforced collagen-based model (COL, reference) and in the 3 reinforced models (SES, 3DP and MEW), at day 3 and day 7. (* = p ≤ 0.05; ** = p ≤ 0.01; *** = p ≤ 0.001).

To study the proliferation potential of the fibroblasts in the tubular collagen-based constructs, the expression of cell proliferation marker Ki-67 was evaluated for each condition, i.e., the non-reinforced collagen-based model (COL, reference) and the three reinforced collagen-based models (SES, 3DP and MEW) by qRT-PCR. Figure 8A shows the results of the gene expression in fold change in relation to the housekeeping gene GAPDH and to the condition of COL at day 3. As shown in Figure 8A, at day 7, all conditions showed a similar expression of Ki-67, and more importantly, an overall higher expression when compared to day 3 (time point, *p* < 0.001). This implies that the fibroblasts continued to proliferate from day 3 to day 7, independently of the condition. Regarding cell viability (Section 3.3(a)), a significantly lower viability for the SES and 3DP-reinforced models was observed compared to COL and MEW but there were no significant differences between day 3 and day 7 when comparing within the same condition. Thus, cell proliferation increased in all conditions from day 3 to day 7, while no difference in cell metabolic activity was observed. This indicates that cells proliferation proceeded although collagen gels were already saturated with cells on day 3.

In addition to their proliferation potential, the ability of the cells to remodel the scaffold and synthesize new ECM are also important factors in the development of an engineered vascular wall model. To evaluate changes in ECM protein synthesis, the expression of two key proteins of the vascular wall were determined. Activated human dermal fibroblasts are known to have a substantial ECM protein expression *in vitro* (Costa-Almeida et al., 2018). Figures 8B,C show the expression of fibrillin-1 and elastin, respectively, for each condition. There is an overall significant upregulation in elastin expression from day 3 to day 7 (time point, *p* = 0.0121). For fibrillin-1, a significant difference was observed between day 3 and day 7 (interaction time point:condition, *p* = 0.0030). More specifically, at day 7, the COL (*p* = 0.004) and the SES (*p* = 0.0036) expressed more fibrillin-1 compared to day 3. The significant differences between conditions are indicated in Figures 8B,C.

The MEW-reinforced model did not show significant differences in the expression of proteins associated with elastic behavior, i.e., elastin and fibrillin-1. Moreover, this condition indicated the earliest induction of gene expression at day 3, with a stable or similar expression observed on day 7. It is hypothesized that the more elastic environment of the MEW-reinforced model (see Section 3.2(b), highest predominant elastic behavior of the different conditions), mimics the physiological environment of the fibroblasts, thus they enter a resting state, during which ECM protein production is not increased.

Recently, several studies have confirmed that matrix stiffness has a significant effect on fibroblast activation in *in vitro* cell cultures (Smithmyer et al., 2014). As an example, Guo et al., 2015 highlighted the importance of the substrate modulus of the scaffold as a key parameter regulating the fibroblasts’ regenerative response. The effect of the substrate’s modulus has also been investigated for the two other vascular cell types, i.e., smooth muscle cells (SMCs) and endothelial cells (ECs). The study of Gabriela Espinosa et al., 2014 stated that SMCs are able to perceive the close presence of elastin and to determine when additional elastin production is indicated. On the other hand, Murikipudi et al., 2013 evaluated the effect of the substrate modulus on the growth and function of matrix-embedded ECs. They concluded that the expression of several common ECM proteins (including collagen IV(α1), collagen IV (α5), fibronectin, *etc.*) by ECs dropped on stiffer substrates, whereas the expression of elastin increased (Murikipudi et al., 2013). Furthermore, Camasao *et al.* (Camasão et al., 2020) also demonstrated how collagen gels showing different elastic environments, designed by introducing elastin-like recombinamers in the gel, resulted in different fibroblast behaviors. Their results showed that collagen hydrogels exhibiting a more elastic environment induced the switch of fibroblasts towards a resting state, along with decreased ECM protein synthesis and proliferation in later stages of collagen gel maturation (Camasão et al., 2020). Achterberg et al., 2014 also demonstrated how matrix stiffness greatly impacts fibroblast behavior. They reported that fibroblasts cultured on substrates mimicking physiological mechanical properties were induced towards a resting state, with a significantly lower expression of ECM proteins, such as elastin and fibrillin-1, compared to fibroblasts cultured on stiffer substrates, which showed an activated state (Achterberg et al., 2014).

From the biological characterization results, we hypothesize that the architecture and mechanical properties of the MEW-reinforced model induced a more physiologically environment, which allowed a rapid switch of fibroblasts from an activated towards a resting state, for which ECM protein synthesis, cell proliferation and metabolic activity remained stable between 3 and 7 days (Lemons et al., 2010). On the other hand, the SES and 3DP-reinforced collagen gels induced a significantly higher cell proliferation and ECM protein synthesis at day 7 compared to day 3, thus maintaining the activated state of fibroblasts (Woodley et al., 2022).

## 4 Conclusion

In this work, the aim was to improve a previously developed cellularized collagen-based vascular wall model by including a tubular polymeric reinforcement scaffold in PCL. Three different processing techniques were compared (i.e., SES, 3DP and MEW), and benchmarked against the non-reinforced cellularized collagen-based model (COL). As shown by SEM imaging, the selected processing technique affects the developed reinforcement scaffold’s architecture on micro- and macro-level. In turn, the scaffold’s architecture (fiber diameter, fiber alignment, pore size) has shown to influence the resulting mechanical and biological properties of the collagen-based model. The tubular, polymeric reinforcements significantly improved the mechanical properties of the reinforced collagen-based model (i.e., initial elastic moduli of 382.05 ± 129.79 kPa, 100.59 ± 31.15 kPa and 245.78 ± 33.54 kPa, respectively for SES, 3DP and MEW at day 7 of maturation) compared to the non-reinforced collagen-based model (ie., 16.63 ± 5.69 kPa). A transition from viscous towards elastic behavior was also observed, showing the highest predominance in elastic behavior for the MEW reinforced model (E_0_/E_e_ ratio of 1.29 ± 0.04). Moreover, the different processing techniques and polymeric tubes’ architecture influenced the cell behavior, in terms of cell attachment, viability, proliferation, ECM protein production and collagen gel compaction. Overall, it can be concluded that 1) the selected processing technique strongly influences the resulting mechanical and biological properties, and 2) the incorporation of a polymeric reinforcement leads to mechanical properties closely resembling those of the native arteries. Based on the results obtained in this study, the MEW reinforcement will be selected for a follow-up study in which a co-culture model will be developed (i.e., endothelial cells, smooth muscle cells and fibroblasts mimicking the three layers of the vascular wall) followed by dynamic maturation.

## Data Availability

The original contributions presented in the study are included in the article/Supplementary Material, further inquiries can be directed to the corresponding authors.

## Supplementary material

Supplementary Figure S1 Visualization of the developed non-reinforced collagen-based model (COL) and the reinforced collagen-based models using 3 different processing techniques (i.e. SES, 3DP and MEW).

Supplementary Figure S2 Schematic overview of the evaluation of the visco-elastic properties through tensile stress relaxation tests using an Instron E1000. **(A)** The ring-shaped samples (L=approx. 4 mm) were positioned on ad hoc made L-shaped grips and tested in a PBS bath at 37°C. **(B,C)** A pre-strain of 5% was applied to the samples, followed by 5 progressive stress relaxation cycles, each consisting of 10% strain ramps and 10 minutes of relaxation. The distance l0 is the initial distance, or the point of zero force calculated with the sample’s original circumference.

## Abbreviations

3D(B)P, 3D (bio)printing; BSA, bovine serum albumin; COL, collagen-based model; DAPI, 4′,6-diamidino-2-phenylindole; DMEM, Dulbecco’s Modified Eagle Medium; EC, endothelial cells; ECM, extracellular matrix; ePTFE, poly (tetrafluoroethylene); (S)ES, (solution) electrospinning; FBS, fetal bovine serum; FDA, U.S. Food and Drug Administration; HC, histochemistry; HDF, human dermal fibroblasts; MEW, melt electrowriting; PBS, phosphate-buffered saline; PCL, poly (ε-caprolactone); PEG, poly (ethylene glycol); PLA, poly (lactic acid); PLCL, poly (L-lactide-co-ε-caprolactone); PLGA, poly (lactic-co-glycolic acid); PU, poly (urethane); PVA, poly (vinyl alcohol); SEM, Scanning Electron Microscopy; SMC, smooth muscle cells; TEBV, tissue engineered blood vessels; vTE, vascular tissue engineering.
