# Supplementary material for: Polymeric reinforcements for cellularized collagen-based vascular wall models: influence of the scaffold architecture on the mechanical and biological properties
Source: Front Bioeng Biotechnol. 2023 Nov 16;11:1285565. doi: 10.3389/fbioe.2023.1285565 (PMC10694796; doi:10.3389/fbioe.2023.1285565)
Supplement: Supplementary file 1 [file Image1.pdf]

Polymeric reinforcements for cellularized collagen-based vascular wall models: Influence of the scaffold architecture on the mechanical and biological properties

Nele Pien[a,b,c], Dalila Di Francesco[a,d], Francesco Copes[a], Michael Bartolf-Kopp[e], Victor Chausse[f], Marguerite Meeremans[c], Marta Pegueroles[f], Tomasz Jüngst[e], Catharina De Schauwer[c], Francesca Boccafoschi[d], Peter Dubruel[b], Sandra Van Vlierberghe[b], Diego Mantovani[a,*]

[a] Laboratory for Biomaterials and Bioengineering, Canada Research Chair Tier I for the Innovation in Surgery, Department of Min-Met-Materials Engineering & Regenerative Medicine, CHU de Quebec Research Center, Laval University, Quebec City, Quebec, Canada

[b] Polymer Chemistry & Biomaterials Group, Centre of Macromolecular Chemistry, Department of Organic and Macromolecular Chemistry, Ghent University, Ghent, Belgium

[c] Faculty of Veterinary Medicine, Department of Translational Physiology, Infectiology and Public Health, Ghent University, 9280 Merelbeke, Belgium

[d] Laboratory of Human Anatomy, Department of Health Sciences, University of Piemonte Orientale "A. Avogadro", 28100 Novara, Italy

[e] Department of Functional Materials in Medicine and Dentistry, Institute of Biofabrication and Functional Materials, University of Würzburg and KeyLab Polymers for Medicine of the Bavarian Polymer Institute (BPI), 97070 Würzburg, Germany

[f] Biomaterials, Biomechanics and Tissue Engineering group, Department of Materials Science and Engineering, Universitat Politècnica de Catalunya, Barcelona, Spain

* Corresponding Author:

Prof. Diego Mantovani, FBSE, FASM, FAIMBE, FCAE

Laboratory for Biomaterials and Bioengineering, Canada Research Chair Tier I for the Innovation in Surgery, Department of Min-Met-Materials Engineering & Regenerative Medicine Division, CHU de Quebec Research Center, Laval University, Pavillon Pouliot, 10655 avenue de la Médecine, 1745G

Québec, Canada, G1V 0A6

Tel: + 1 418-656-2160 ext 406270 or + 1 418 717 0828

E-mail: Diego.Mantovani@gmn.ulaval.ca , website www.lbb.ulaval.ca

Supplementary Information

Supplementary Information includes two figures:

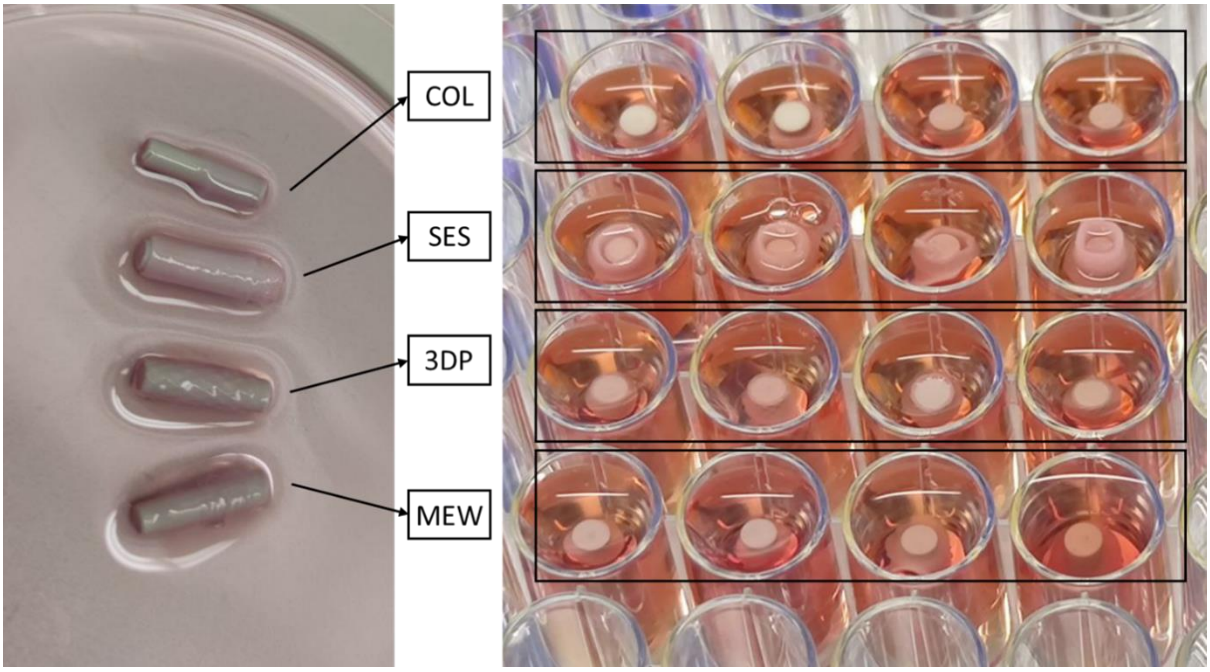

Figure S.1. Visualization of the developed non-reinforced collagen-based model (COL) and the reinforced collagen-based models using 3 different processing techniques (i.e. SES, 3DP and MEW).

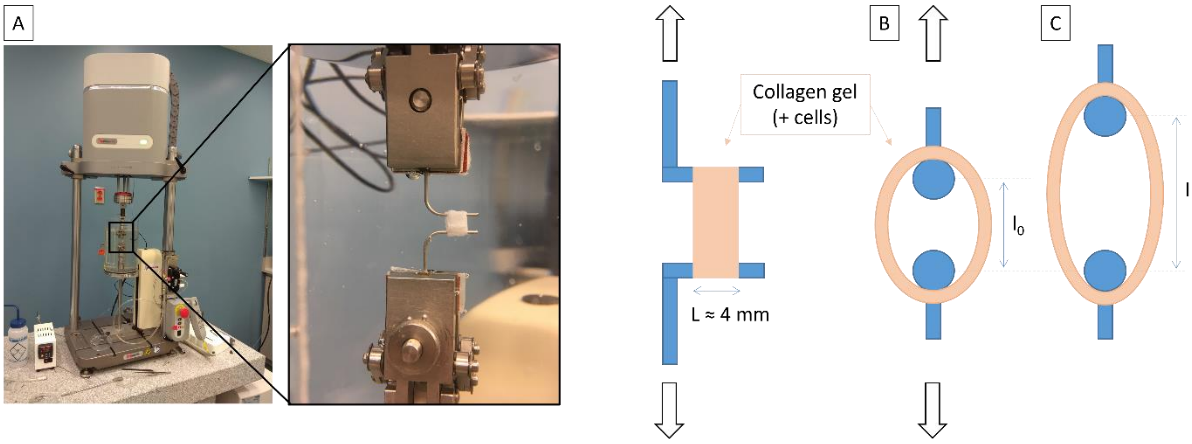

Figure S.2. Schematic overview of the evaluation of the visco-elastic properties through tensile stress relaxation tests using an Instron E1000. (A) The ring-shaped samples (L = approx. 4 mm) were positioned on ad hoc made L-shaped grips and tested in a PBS bath at 37°C. (B-C) A pre-strain of 5% was applied to the samples, followed by 5 progressive stress relaxation cycles, each consisting of 10% strain ramps) and 10 minutes of relaxation. The distance l_0 is the initial distance, or the point of zero force calculated with the sample's original circumference.
